# Transcriptome Comparison Analysis of *Ostrinia furnacalis* in Four Developmental Stages

**DOI:** 10.1038/srep35008

**Published:** 2016-10-07

**Authors:** Tiantao Zhang, Kanglai He, Zhenying Wang

**Affiliations:** 1 State Key Laboratory for the Biology of Plant Diseases and Insect Pests, Institute of Plant Protection, Chinese Academy of Agricultural Sciences, No. 2 West Yuanmingyuan Road, Beijing 100193, China

## Abstract

The Asian corn borer, *Ostrinia furnacalis*, is one of the most destructive pests of maize and causes huge losses in maize yield each year. In order to characterize the different developmental stages, a high-throughput sequencing platform was employed to perform *de novo* transcriptome assembly and gene expression analysis for the egg, larva, pupa and adult stages. Approximately 185 million reads were obtained, trimmed, and assembled into 42,638 unigenes with an average length of 801.94 bp and an N50 length of 1,152 bp. These unigene sequences were annotated and classified by performing Gene Ontology (GO), Cluster of Orthologous Groups (KOG) and Kyoto Encyclopedia of Genes and Genomes (KEGG) functional classifications. Comparison of the gene expression profiles of the two transitional stages revealed dramatic differences. Some differentially expressed genes are associated with digestion, cuticularization olfactory recognition and wing formation as well as growth and development. In total, 12 putative insect development-related genes were identified. Real-time quantitative PCR (RT-qPCR) results and sequencing based on relative expression levels of randomly selected genes confirmed these expression patterns. These data represent the most comprehensive transcriptomic resource currently available for *O. furnacalis* and will facilitate the study of developmental pathways, cuticularization, wing formation and olfactory recognition.

Corn (*Zea mays* L.) is ranked first crop plants in terms of planting area and total yield^1^. The lepidopteran Asian corn borer (ACB), *Ostrinia furnacalis* (Guenée), is a common borer pest throughout eastern China and Southeast Asia, as well as the Pacific and Australasia, and causes serious damage to corn, sorghum, millet and cotton^2^. The first and second larval stages easily damage maize during the whorl leaf stage and then attack the stem, ears and cobs. The larvae also feed on silks and kernels, causing ear rot, increased mycotoxin contamination and reduced grain quality. Annual yield losses caused by ACB are estimated to be 6 to 9 million tons^3^.

Several factors contribute to the status of *O. furnacalis* as a devastating pest species, including adaptation to many host crops, high fecundity, a high capacity to evolve resistance to Bt^4^,^5^ and other pesticides and the ability to synchronize its life cycle through diapause^6^. Diapause is an endocrine-controlled mechanism that allows insects to escape unfavorable conditions, such as overwintering or drought, during certain stages of their life history^7^. Insect diapause is primarily determined by photoperiodic responses^8^. The *O. furnacalis* moth is a typical long-day-length species; to adapt to winter, the larvae undergo a facultative diapause and respond to short-day conditions during the autumn^9^. This insect overwinters in the form of full-grown larvae in plant stalks, and as the temperature increases in the following year, the larvae become pupae and emerge as moths. In the Huang-Huai region of China, multiple generations of *O. furnacalis* are initiated within the span of a year, and the larvae of the second and third generations cause the majority of the damage to maize; however, only two generations are produced in the Northeast region, while *O. furnacalis* produces up to seven generations in the south of China.

The ACB moth is important for both basic and applied biology research, specifically evolutionary biology, chemical ecology and pest management. The majority of *Ostrinia* species employ varying ratios of (E)-11- and/or (Z)-11-tetradecenyl acetate (E11- and Z11-14: OAc) as sex pheromone components. However, the ACB is unique within the genus *Ostrinia* because it has evolved to use a pheromone blend involving a shift in the position of the double bonds, resulting in E12- and Z12-tetradecenyl acetate^10^,^11^.

The rapid development of next-generation sequencing, such as Illumina sequencing, has provided cost-effective methods for the transcriptomic characterization of insect species that lack a fully-sequenced genome. Many insects have been sequenced, including the ACB^12^,^13^,^14^,^15^ and its sibling species, the European corn borer^16^,^17^. The deeper sequencing coverage of Illumina sequencing combined with de novo transcriptome assembly will facilitate the characterization of genes in the absence of a reference genome. We used Illumina HiSeq 2000 to generate a substantial dataset of transcript reads at different stages of *O. furnacalis* development. The primary aims of this study were to compare gene expression levels in different developmental stages and create a database of molecular information to identify genes related to ACB development.

## Materials and Methods

### Insect Rearing

*O. furnacalis* larvae were reared on an artificial diet at the Institute of Plant Protection, Chinese Academy of Agricultural Sciences, Beijing, China. The larvae were kept indoors at a temperature of 27 ± 1 °C with an L:D photoperiod of 16:8 h and 70–80% relative humidity. Eggs, 3^rd^ stage larvae, pupae and three-day-old moths were collected and immediately placed in liquid nitrogen, then transferred to a −80 °C freezer until use.

### RNA Isolation, cDNA Library Preparation and Illumina Sequencing for Transcriptome Analysis

Total RNA from eggs, larvae, pupae and moths was extracted separately using TRIzol reagent (Invitrogen, Carlsbad, CA, USA) according to the manufacturer’s instructions, and cDNA library construction and Illumina sequencing were subsequently performed at Gene Denovo Biotechnology Co., Ltd, China. Briefly, approximately 20 μg of pooled total RNA samples from individual *O. furnacalis* developmental stages were digested with DNase I (Sigma, USA), enriched for mRNA via purification with oligo(dT) magnetic beads and fragmented into fragments of 100–400 bp. First-strand cDNA was synthesized using SuperScript II and random primers. Subsequently, second-strand cDNA was synthesized using DNA polymerase I and RNaseH. The cDNAs were fragmented, end-repaired, and ligated with library-specific barcoded adaptors. Library fragments were PCR-amplified for a minimum number of cycles to avoid normalization for downstream quantitative gene expression analyses. Amplified products were purified with a QIAGEN MiniElute PCR Purification Kit (Qiagen, Venlo, Netherlands), and approximately equal molar proportions of male and female indexed libraries were sequenced in a single flow cell of an Illumina HiSeq 2000 sequencing machine.

### Assembly and Functional Gene Annotation

The raw data outputs from the Illumina equipment were trimmed for quality scores (q) < 20, and adaptor, N < 10% and low-quality reads were removed to obtain high-quality, clean reads. The clean reads were assembled using Trinity software (http://trinityrnaseq.sourceforge.net/) to produce unigenes. Subsequently, overall unigenes were analyzed and annotated. Unigenes were aligned with the Nr, Nt, SwissProt, and TrEMBL databases using BLAST with a cut-off E-value of 10^−5^. Functional gene annotations were collected for all unigene sequences ≥150 bp using Blast2GO^18^. Initial searches of the National Center for Biotechnology Information (NCBI) non-redundant (Nr) protein database were conducted using the BLASTx algorithm, followed by the collection of Gene Ontology (GO) terms from the GO database and the retrieval of Kyoto Encyclopedia of Genes and Genomes (KEGG) pathway designations.

### Differential Gene Expression and Trend Analysis

Differential unigene abundances were determined by performing independent alignments of short reads from four libraries against the set of *O. furnacalis* lifetime unigenes using Blat software. Reads per kb per million reads (RPKM) were calculated as: RPKM = (1000000*C)/(N*L/1000), where C is the number of mappable reads in specific unigenes, N is the total number of reads mapped to unigenes in a particular sample, and L is the length of the unigene^19^. To analyze the expression profiling of eggs, larvae, pupae and moths based on RPKM values, Short Time-series Expression Miner (STEM) software (http://www.cs.cmu.edu/~jernst/stem) was used to compare the trends exhibited in these four stages^20^. P-values correspond to the differential gene expression test, which was performed to analyze all trends in these four stages.

## Results

### Illumina Sequencing and Data Assembly

To further understand the molecular mechanisms involved in the developmental changes during *O. furnacalis* life stages, cDNA libraries from eggs (accession number: SRX1613687), larvae (accession number: SRX1613690), pupae (accession number: SRX1613689) and moths (accession number: SRX1601946) were sequenced using the Illumina HiSeq^TM^ 2000 sequencing platform. A total of 185,436,912 reads were generated and assembled into 42,638 unigenes from four cDNA libraries (Tables 1 and 2) with an average length of 801.94 bp and an N50 length of 1,152 bp (Table 2). The cDNA libraries produced a total of 36,202,888 clean reads, which represent the majority of the data, and the Q20 score was >95%. Quality checks and unigene assembly were carried out using Trinity software.

### Annotation of Predicted Proteins

**Figure 1:**
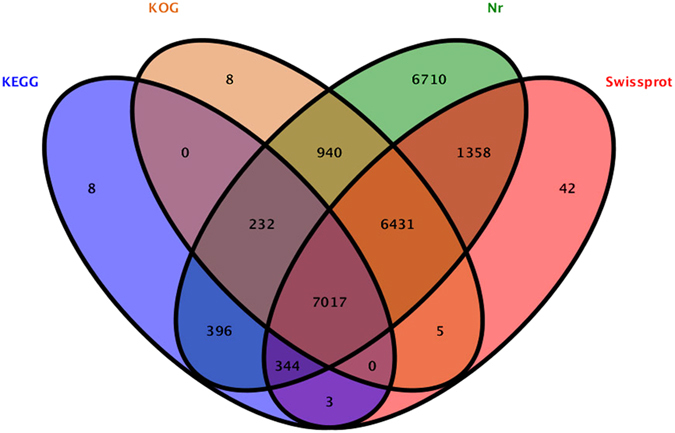
Comparison of unigene annotation in the Nr, SwissProt, KOG and KEGG databases.

**Figure 2:**
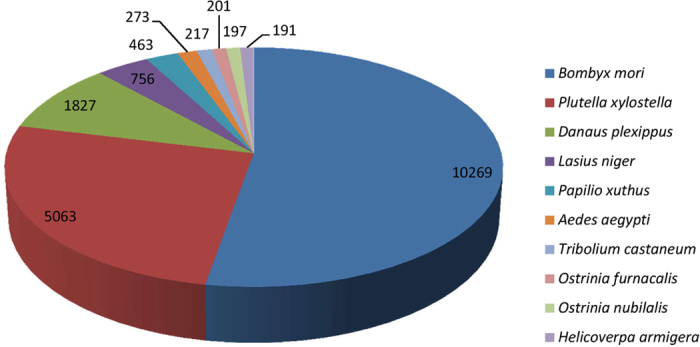
The top-ten species distribution of the BLASTX results. Unigenes were aligned with the NCBI-Nr protein database with a cutoff E value < 10^−5^. Different colors represent different species.

The unigenes obtained for *O. furnacalis* were annotated as hypothetical proteins using BLASTX tools based on various protein databases^21^. A total of 23,494 unigenes (approximately 55.1%) were annotated based on four main databases (Table 3). Approximately 23,428 unigenes (54.95%) were matched in the non-redundant (Nr) database, including 6,700 unigenes that specifically matched this database. Additionally, 15,200 unigenes (approximately 35.65%) had significant matches in the SwissProt database, including 42 unigenes that only matched this database (Fig. 1). Approximately 14,633 (34.32%) and 8,000 (18.76%) unigenes had specific matches in the KOG and KEGG databases, respectively. The top 10 species distributions are shown in Fig. 2. Approximately 10,269 unigenes were annotated to 10 top-hit insect species. *Bombyx mori* and *Plutella xylostella* were the 2^nd^ top-hit species, with 10,269 and 5,063 annotated genes, respectively. The other top-hit species were *Danaus plexippus*, *Lasius niger*, *Papilio xuthus*, *Aedes aegypti*, *Tribolium castaneum*, *O. furnacalis*, *O. nubilalis*, and *Helicoverpa armigera*. All alignment species are shown in Table S1.

### GO, KOG and KEGG Classifications

The functional classification of *O. furnacalis* unigenes was predicted by performing GO, KOG and KEGG analyses. A total of 11,511 unigenes were allocated to three specific GO categories: cellular component, biological process and molecular function. Approximately 52,327 unigenes were putatively identified as having GO functions, including 26,631 sequences (50.89%) at the biological process level, 14,347 sequences (27.42%) at the cellular component level and 11,349 sequences (21.69%) at the molecular function level. In total, 54 categories were subdivided from the primary categories: 25 categories for biological process, 16 categories for cellular component and 13 categories for molecular function.

**Figure 3:**
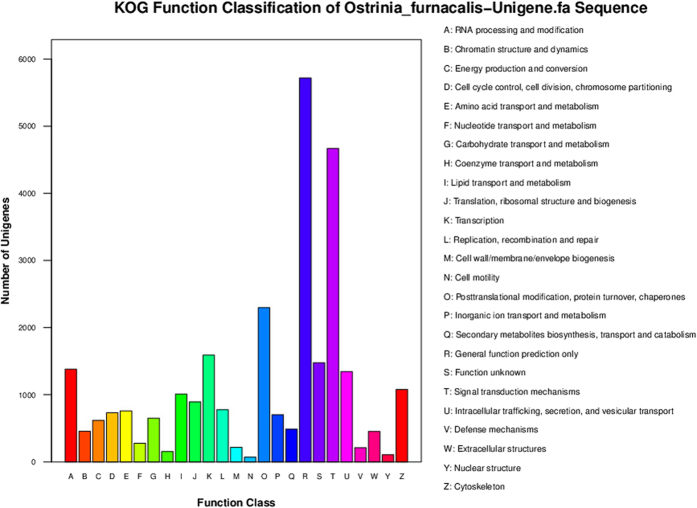
KOG functional classification of the unigenes. The names of each class definition (A to Z) are provided on the right side of the figure.

**Figure 4:**
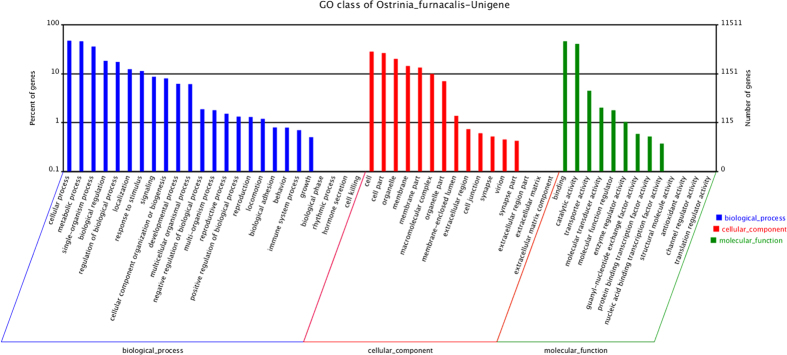
GO unigene categories. The unigenes were annotated in three main categories: biological process (blue), cellular component (red) and molecular function (green). The left side of the y-axis represents the percentage of a specific category of genes for a main category. The right side of the y-axis represents the number of genes in a category.

For KOG functional classification, approximately 14,633 unigenes were annotated for 28,129 functions involved in 25 KOG categories (Fig. 3). Among them, the largest group was the “General function prediction” (5,717 genes, 20.32%), followed by the large groups (i.e., >1000 genes) “Signal transduction mechanisms” (4,668 genes, 16.59%), “Posttranslational modification, protein turnover, chaperones” (2,297 genes, 8.17%), “Transcription” (1,590 genes, 5.65%), “RNA processing and modification” (1,380 genes, 4.91%), “Intracellular trafficking, secretion, and vesicular transport” (1,347 genes, 4.79%), “Cytoskeleton” (1,078 genes, 3.83%) and “Lipid transport and metabolism” (1,009 genes, 3.59%). Intermediate groups (i.e., 500-1,000 genes) predicted by KOG included “Translation, ribosomal structure and biogenesis” (895 genes, 3.18%), “Replication, recombination and repair” (778 genes, 2.77%), “Amino acid transport and metabolism” (759 genes, 2.70%), “Cell cycle control, cell division, chromosome partitioning” (731 genes, 2.60%), “Inorganic ion transport and metabolism” (701 genes, 2.49%), “Carbohydrate transport and metabolism” (650 genes, 2.31%), and “Energy production and conversion” (617 genes, 2.19%). The smallest groups (1–499 genes) predicted by KOG included “Secondary metabolites biosynthesis, transport and catabolism” (488 genes, 1.73%), “Chromatin structure and dynamics” (457 genes, 1.62%), “Extracellular structures” (453 genes, 1.61%), “Nucleotide transport and metabolism” (278 genes, 0.99%), “Cell wall/membrane/envelope biogenesis” (216 genes, 0.77%), “Defense mechanisms” (211 genes, 0.75%), “Coenzyme transport and metabolism” (154 genes, 0.54%), “Nuclear structure” (106 genes, 0.75%) and “Cell motility” (72 genes, 0.26%). There were 1,477 genes (5.25%) with unknown functions (Fig. 4).

*O. furnacalis* unigene sequences that mapped to the reference canonical pathways in the KEGG database were analyzed. We specifically assigned 14,947 unigene sequences to 236 KEGG pathways; 1,364 unigenes were involved in metabolic pathways, and these pathways played dominant roles in the following pathways: RNA transport, insect hormone biosynthesis, olfactory transduction, ubiquitin-mediated proteolysis and others (Table S2).

### Differential Gene Expression among *O. furnacalis* Developmental Stages

To compare the differential expression levels of genes between the major developmental stages, up- and down-regulated gene numbers were calculated between every pair of *O. furnacalis* life-stages that represented specific transitional developmental stages in holometabolic insects (eggs and larvae, larvae and pupae, pupae and adults, adults and eggs). The top 10 genes for each comparison are listed in Table S3. Based on analyses of the pathways, annotation and the reported literature, 12 insect development-related genes were identified and submitted to the NCBI database.

In total, 19,401 different genes demonstrating significant expression profile changes in the hatching-to-larva process, including 8,168 up-regulated genes and 11,233 down-regulated genes, were obtained in the comparative analysis between eggs and larvae. Four of the ten most up-regulated genes in the egg transcriptome had homology to *B. mori* genes of neuropeptide-like gene 4, mucin-5AC-like gene, salivary cysteine-rich peptide precursor gene, and cuticular protein gene RR-1 motif 20 isoform X1. Four genes with unknown functions were found to be similar to GJ14248 of *Drosophila virilis*, CG12017 of *Papilio xuthus*, uncharacterized protein gene LOC105388107 of *Plutella xylostella* and hypothetical protein KGM_22714 of *Danaus plexippus*. Two genes had putative functions as a secreted protein of *Papilio polytes* and a serine protease of *Bombyx mandarina*. Among the ten most down-regulated genes, three had predicted functions as the proteoglycan, mucin and segmentation proteins of *P. xylostella*. Two genes putatively matched the pair-rule protein and leucine-rich repeat extensin-like protein 5 of *B. mori*. Two genes matched the hypothetical protein KGM_13352 and glucose dehydrogenase of *D. plexippus*. One gene was predicted to function as the epidermal growth factor receptor of *Bicyclus anynana* and one as the histone 2A variant of *Spodoptera frugiperda* (Table S3).

In the larva-to-pupation process, comparison between the larval and pupal transcriptomes revealed 20,130 significantly differentially expressed genes, including 6,251 up-regulated genes and 13,879 down-regulated genes in the larvae library. Three of the top ten up-regulated genes were aligned with *B. mori*, with the predicted functions of larval cuticle protein LCP-22 precursor, zonadhesin-like isoform X2 and uncharacterized protein LOC101735588. Two genes matched with two hemipteran insects (*Monomorium pharaonic* and *Linepithema humile*), with functions as collagen alpha-5(IV) and mucin-19. Furthermore, two genes matched with two Noctuidae insects (*Spodoptera exigua* and *Helicoverpa armigera*), with MG17 and Niemann-Pick type C2 protein functions. Two genes that matched closely related species (*O. nubilalis* and *Cnaphalocrocis medinalis*) were predicted to have the functions of a trypsin-like serine protease and a chemosensory protein. Only one gene was similar to the adhesive plaque matrix protein in *Saccoglossus kowalevskii*. The top ten hits for down-regulated genes in the larvae library included the following predicted functional genes: the glucose dehydrogenase gene and the flocculation protein gene in *B. mori*; the uncharacterized protein gene LOC105388107, the mucin-17 gene and the formin-J-like gene in *P. xylostella*; the CG12017 gene in *P. xuthus*; the peritrophin type-A domain protein 3 gene in *Danaus plexippus*; the seminal fluid protein gene CSSFP002 in *Chilo suppressalis*; and the collagen alpha-2(I) gene in *Columba livia* (Table S3).

Differentially expressed genes were observed before and after ACB emergence. A total of 18,833 significantly expressed genes (including 8,752 up-regulated genes and 10,081 down-regulated genes) were obtained in the pupae library. Among the top ten gene hits, five genes matched with *P. xylostella* with functions of the nose resistant to fluoxetine protein 6-like gene, the uncharacterized protein gene LOC105384628, the uncharacterized protein gene LOC105388107, the probable ATP-dependent helicase gene PF08_0048 and the troponin C gene. The predicted functions of the other five top-ten genes included the nuclear-pore anchor-like gene (*Bombyx mori*), the insect intestinal mucin 4 gene (*Danaus plexippus*), the retinol-binding protein gene (*Papilio xuthus*), the chitin binding domain 3 protein gene (*Mamestra configurata*) and the general odorant-binding protein 3 gene (*Cnaphalocrocis medinalis*). Among the top ten down-regulated genes in the pupal transcriptome, five genes had predicted functions as the larval cuticle protein LCP-17 precursor gene (*B. mori*), the GL14840 gene (*Drosophila persimilis*), the chitin deacetylase 2 gene (*Mamestra configurata*), the chymotrypsin-like serine protease gene (*O. nubilalis*) and the pancreatic triacylglycerol lipase-like gene (*B. mori*). The other genes with predicted functions were the trypsin serine protease gene (*O. furnacalis*), the lipase gene (*H. armigera*), the trypsin-like serine protease gene (*O. nubilalis*), and the mucin-19-like gene (*Linepithema humile*) (Table S3).

**Figure 5:**
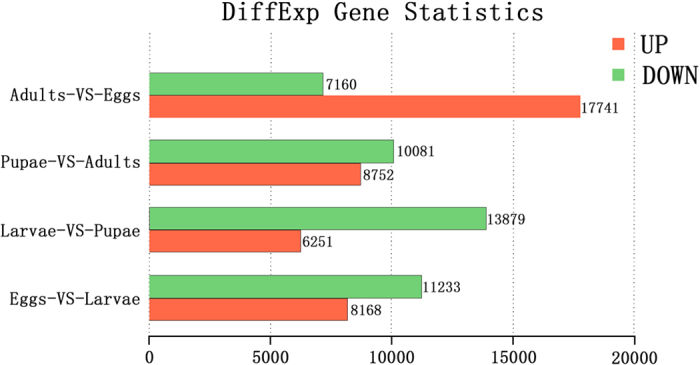
Comparison of the numbers of unigenes in different developmental stages in *Ostrinia furnacalis*. Up-regulated unigenes are marked in red, and down-regulated unigenes are marked in green.

When differential gene expression was assessed between the adult and egg transcriptomes, 24,901 genes exhibiting significantly differential expression were identified. Of these genes, 17,741 were up-regulated and 7,160 were down-regulated in the adult transcriptome (Fig. 5). Five up-regulated genes matched the uncharacterized protein genes KIAA0753 (*Erinaceus europaeus*), LOC105381897 and LOC105387542 (*P. xylostella*) and LOC101744818 and LOC101741925 (*B. mori*), and five up-regulated genes had predicted functions, specifically the repetitive proline-rich cell wall protein 2-like gene (*Chrysochloris asiatica*), the hypothetical protein gene KGM_11707 (*Danaus plexippus*), the flexible cuticle protein 12-like gene (*P. xylostella*), the Kunitz protein 8 gene (*Echinococcus granulosus*) and the histone H2A sperm-like gene (*P. xylostella*). Interestingly, 7 down-regulated genes matched to the cuticle protein genes in *B. mori*, *P. xuthus* and *P. xylostella*. One gene was similar to the JH-inducible protein gene in *Danaus plexippus*, one gene matched with the neuropeptide-like gene 4 in *B. mori* and one gene was similar to the chemosensory protein gene in *Cnaphalocrocis medinalis* (Table S3).

### Trend Analysis

**Figure 6:**
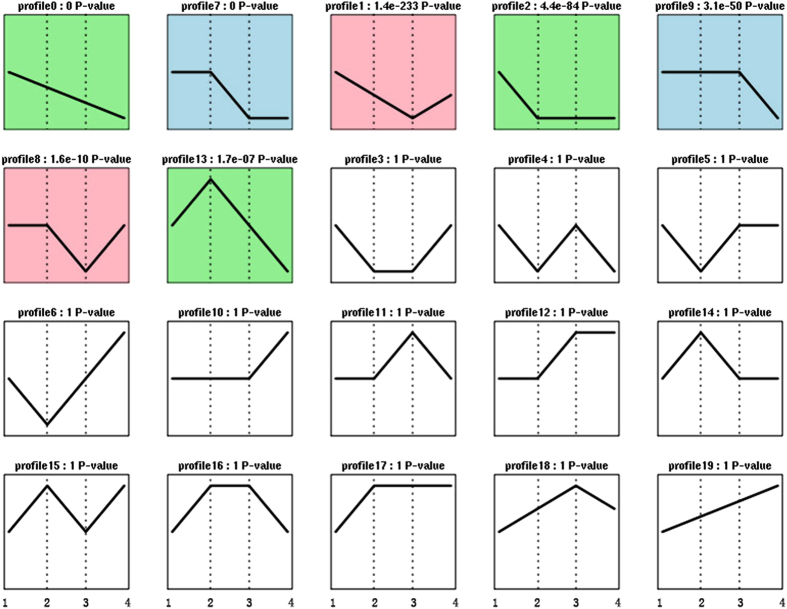
DEG expression profiles for the four developmental stages based on P-values. Colored squares represent significant enrichment, and non-colored squares represent no significant enrichment. “1” represents the egg stage, “2” represents the larval stage, “3” represents the pupal stage, and “4” represents the adult stage.

To examine the expression profiles of the 27,986 differentially expressed genes (DEGs), the expression data from eggs, larvae, pupae and adults was clustered into 15 profiles by Short Time-series Expression Miner software (STEM), in which 27,986 were clustered into 7 profiles (p-value ≤ 0.05) (Fig. 6), including genes demonstrating down-regulated patterns (Profile 0), comprising 4,233 DEGs. Profile 7, which contained 3,467 DEGs, showed down-regulation from the larvae to the pupae stage and maintained balance during the transitions from eggs to larvae and pupae to adults. Similar to profile 7, profile 8, which included 1,868 DEGs, demonstrated up-regulation during the emergence stage. Profile 1 showed down-regulation from eggs to pupae and then up-regulation during the transition to adults; this profile included 2,217 DEGs. Profile 2 comprised 2,308 DEGs and showed down-regulation during the hatching stage (eggs to larvae), then did not change during the other stages. Profile 9 contained 1,908 DEGs and showed no changes prior to pupation, then exhibited down-regulation during the adult stage. Profile 13 contained 1,203 DEGs and showed up-regulation during the larval stage, then was down-regulated during the adult stage.

### RT-qPCR Analysis

**Figure 7:**
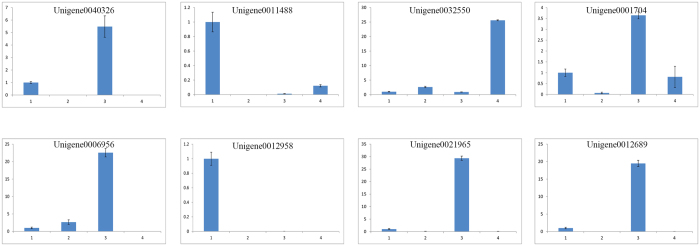
RT-qPCR analysis of eight randomly selected genes to confirm expression patterns indicated by sequencing. Three technical replicates were performed for each of the three biological replicates. The height of each bar chart represents the mean average of sample-specific 2^−ΔΔCt^ values. “1” represents the egg stage, “2” represents the larval stage, “3” represents the pupal stage, and “4” represents the adult stage.

To confirm the accuracy and reproducibility of the transcriptome analysis results, RT-qPCR analyses were performed on eight randomly selected genes: Unigene0040326, Unigene0011488, Unigene0032550, Unigene0001704, Unigene0006956, Unigene0012958, Unigene0021965, and Unigene0012689. β-actin served as the reference gene for RT-qPCR normalization. The analyzed results in Fig. 7 supported the differentially expressed unigenes (DEU). Unigene0040326, Unigene0040326, Unigene0006956, Unigene0021965 and Unigene0012689 were primarily expressed in pupae, whereas Unigene0011488 and Unigene0012958 were primarily expressed in eggs. Only Unigene0032550 was primarily expressed in adults. The high confirmation rate of the unigenes indicated the reliability of the transcriptome data.

## Discussion

In this study, we compared the transcriptomes of four developmental stages (eggs, larvae, pupae and adults) of the maize pest *O. furnacalis*. A profound understanding of the molecular mechanisms regulating pest life cycles and development at each stage may aid in the control of this pest by facilitating the development of more sustainable and environmentally-friendly approaches. These results significantly increase the molecular resources available for the study of other insect pests and provide a framework to understand changes at the gene expression level during insect development. We assembled transcriptomes from all four stages and obtained a total of 42,638 unigenes with an average length of 801.94 bp. Approximately 23,494 sequences from this insect were annotated based on four main databases. Approximately half of the total unigenes had not been previously annotated; thus, our Illumina sequencing depth and analysis in this study represent improvements over previous reports^22^,^23^. Many assembled unigenes were not significantly matched with available databases due to their short sequences or because they represented significantly novel genes. Seven of the top 10 annotated unigenes belong to Lepidopteran, highlighting the conservation of many genes in Lepidopteran. Although we lack the full genome information for *O. furnacalis*, annotation of the four developmental stages transcriptomes indicated a high proportion of functional genes for this insect.

The ACB exhibits the typical developmental characteristics of holometabolous metamorphosis, and insects develop from eggs to five-stage larvae, then to pupae, and eventually emerge as moths. Moths lay eggs and begin a new life cycle. During the transition from eggs to larvae, 8,168 up-regulated genes and 11,233 down-regulated genes were identified. Among the ten most up-regulated genes, many were related to immunity, digestion and cuticularization, such as the salivary cysteine-rich peptide precursor gene and the cuticular protein gene RR-1^24^,^25^,^26^. The cuticular protein genes have also been identified in the larval stages of *Athetis lepigone* and other insects, which indicates these protein genes are ubiquitous in insects^23^. Insect cuticles are composed of cuticular protein and chitin, which not only support and maintain the physical structure of the organism but also serves as natural barriers against external adverse impacts^27^. During the transition from egg to larva, cuticle composition and performance rapidly change due to alterations in cuticular proteins; these changes facilitate the performance of cuticle-based structures^28^. Our results provide useful information for further studies investigation cuticular proteins and the cuticle. Furthermore, the segmentation protein was down-regulated in larvae, suggesting this gene functions in cell mitosis. When larvae and pupae were compared, one gene encoding Niemann-pick type C2 protein was identified; this protein is an essential carrier protein for cholesterol and may function in chemical communication from late endosomes and lysosomes to other cellular organelles^29^. The putative peritrophin type-A domain protein 3 gene was also identified in the transcriptome. The peritrophic membrane (PM) is a film-like structure that separates food from the insect midgut tissue. It protects the epithelium against food abrasion and microorganisms and has other functions involved in enzyme compartmentalization^30^. The peritrophin type-A domain protein 3 genes may also carry out additional functions during feeding stages. At the adult stage, the retinol-binding protein gene, an olfactory related gene, a general odorant-binding protein gene and the troponin C gene were identified. These genes are important for moth flight and the ability of moths to seek mates and hosts, particularly the general odorant-binding protein (GOBP) and the troponin C gene. GOBP is small water-soluble protein that transports hydrophobic odorants through the aqueous sensillar lymph and plays an important role in insect chemoreception^31^,^32^. The troponin C gene may carry out functions for moth flight. Moth flight behavior is dependent on the wings, which are moved by resonant changes in the shape of the thorax produced by indirect flight muscles; muscle contraction is regulated by changes in the Ca^2+^ concentration in the fibers. Troponin C, which has a single Ca^2+^-binding site near the C-terminus, regulates these muscles in many insect genera^33^.

Based on the analyzed pathways, annotations and reported literature, 12 insect development-related genes were identified based on the comparison of the transcriptomes at different stages. Many genes participate in insect developmental processes. Various genes, such as segmentation genes, control cellular identity during early pattern formation in *Drosophila*^34^. Unigene0011488 was annotated as segmentation protein fushi tarazu, which has putative function in development at the blastoderm stage. Furthermore, this gene is transiently expressed in a specific subset of neuronal precursor cells, neurons (such as aCC, pCC, RP1, and RP2), and glia in the developing central nervous system (CNS), suggesting a role in the transformation of neuronal identity^34^. Unigene0012958 was annotated as an epidermal growth factor receptor; Unigene0001331, Unigene0016506, Unigene0001330, Unigene0021965 and Unigene0026727 were annotated as cuticular proteins. These two gene types have previously been reported to function in epidermal growth^35^, cuticle sclerotization and adult molting^36^. The neuropeptide-like 4 gene (Unigene0033676) is expressed uniquely during diapause, suggesting a potential role in initiating and maintaining diapause in the flesh fly^37^. Other genes, such as chemosensory protein (Unigene0032550) and chitin deacetylase 2, have functions related to larval development^38^ and developmental timing delays^39^. All identified genes represent candidate genes for future studies.

This study constructed four transcriptome libraries and generated much-needed genetic and genomic resources for the investigation of *O. furnacalis*. Future analyses of genes related to insect development or death will be useful for pest control. Additionally, the abundance of unigenes and expression data derived from this study will supply information regarding the identification of genes involved in *O. furnacalis* development.

## Additional Information

**How to cite this article**: Zhang, T. *et al*. Transcriptome Comparison Analysis of *Ostrinia furnacalis* in Four Developmental Stages. *Sci. Rep*. **6**, 35008; doi: 10.1038/srep35008 (2016).

## Supplementary Material

Supplementary Information

## Figures and Tables

**Table 1:** Summary of statistical data for the transcriptomes of *O. furnacalis* developmental stages.

Sample	Total Raw Reads	Total Clean Reads	GC%	Q20 (%)	Low quality (%)
Eggs	38,095,520	36,202,888	49.55%	96.26%	4.93%
Larvae	48,464,578	46,136,682	49.59%	96.40%	4.76%
Pupae	42,569,634	40,411,990	51.39%	96.15%	5.02%
Adults	66,287,998	62,685,352	49.76%	95.78%	5.42%
All	195,417,730	185,436,912			

**Table 2:** Summary of assembly statistics for the different developmental stages of *O. furnacalis*.

Num Genes	GC percentage	N50	Max length	Min length	Average length	Total assembled bases
42,638	43.87%	1,152	18,095	229	801.94	34,193,208

N50 is the 50% length of all unigenes.

**Table 3:** Functional annotations of total unigenes in *O. furnacalis*.

Sequence File	Nr	SwissProt	KOG	KEGG	ALL
unigenes	23,428	15,200	14,633	8,000	23,494

Nr: non-redundant database; SwissProt: Swiss Protein database; KOG: Clusters of Orthologous Groups of proteins; KEGG: Kyoto Encyclopedia of Genes and Genomes.
