# Supplementary material for: Transcriptome Comparison Analysis of Ostrinia furnacalis in Four Developmental Stages
Source: Sci Rep. 2016 Oct 7;6:35008. doi: 10.1038/srep35008 (PMC5054526; doi:10.1038/srep35008)
Supplement: Supplementary Information [file srep35008-s1.doc]

Supplementary tables

**Transcriptome Comparison Analysis of *Ostrinia furnacalis* in Four Developmental Stages**

Tiantao Zhang, Kanglai He & Zhenying Wang*

State Key Laboratory for the Biology of Plant Diseases and Insect Pests, Institute of Plant Protection, Chinese Academy of Agricultural Sciences, No. 2 West Yuanmingyuan Road, Beijing 100193, China

*Corresponding author:

E-mail addresses: zhtiantao@163.com; zywang@ippcaas.cn

Tel: +86 10 62815945; Fax: +86 10 62815945

**Table S1** Species distribution statistics in *Ostrinia furnacalis* transcriptomes

| **Species** | **Unigene Num** |
| --- | --- |
| ***Bombyx mori*** | 10269 |
| ***Plutella xylostella*** | 5063 |
| ***Danaus plexippus*** | 1827 |
| ***Lasius niger*** | 756 |
| ***Papilio xuthus*** | 463 |
| ***Aedes aegypti*** | 273 |
| ***Tribolium castaneum*** | 217 |
| ***Ostrinia furnacalis*** | 201 |
| ***Ostrinia nubilalis*** | 197 |
| ***Helicoverpa armigera*** | 191 |
| ***Chilo suppressalis*** | 183 |
| ***Papilio polytes*** | 143 |
| ***Anopheles gambiae str. PEST*** | 129 |
| ***Manduca sexta*** | 121 |
| ***Zootermopsis nevadensis*** | 107 |
| ***Bacillus*** | 105 |
| ***Drosophila melanogaster*** | 98 |
| ***Cnaphalocrocis medinalis*** | 96 |
| ***Stegodyphus mimosarum*** | 81 |
| ***Spodoptera frugiperda*** | 73 |
| ***Heliconius erato*** | 68 |
| ***Anopheles sinensis*** | 64 |
| ***Camponotus floridanus*** | 60 |
| ***Acyrthosiphon pisum*** | 60 |
| ***Spodoptera litura*** | 60 |
| ***Microplitis demolitor*** | 54 |
| ***Agrotis segetum*** | 53 |
| ***Athalia rosae*** | 50 |
| ***Diaphorina citri*** | 49 |
| ***Antheraea mylitta*** | 48 |
| ***Culex quinquefasciatus*** | 47 |
| ***Spodoptera exigua*** | 47 |
| ***Drosophila mojavensis*** | 46 |
| ***Harpegnathos saltator*** | 45 |
| ***Drosophila willistoni*** | 45 |
| ***Orussus abietinus*** | 37 |
| ***Acromyrmex echinatior*** | 36 |
| ***Galleria mellonella*** | 36 |
| ***Cerapachys biroi*** | 36 |
| ***Megachile rotundata*** | 35 |
| ***Drosophila grimshawi*** | 35 |
| ***Hydra vulgaris*** | 34 |
| ***Spodoptera littoralis*** | 34 |
| ***Nasonia vitripennis*** | 33 |
| ***Vollenhovia emeryi*** | 32 |
| ***Drosophila ananassae*** | 32 |
| ***Metaseiulus occidentalis*** | 32 |
| ***Loxostege sticticalis*** | 31 |
| ***Drosophila pseudoobscura pseudoobscura*** | 31 |
| ***Trichuris trichiura*** | 30 |
| ***Anopheles gambiae*** | 30 |
| ***Mamestra configurata*** | 28 |
| ***Solenopsis invicta*** | 28 |
| ***Saccoglossus kowalevskii*** | 28 |
| ***Drosophila virilis*** | 28 |
| ***Drosophila yakuba*** | 26 |
| ***Drosophila persimilis*** | 25 |
| ***Biston betularia*** | 25 |
| ***Musca domestica*** | 24 |
| ***Trichinella spiralis*** | 24 |
| ***Anopheles darlingi*** | 24 |
| ***Heliconius melpomene*** | 23 |
| ***Antheraea pernyi*** | 22 |
| ***Wasmannia auropunctata*** | 22 |
| ***Strongylocentrotus purpuratus*** | 21 |
| ***Linepithema humile*** | 21 |
| ***Crassostrea gigas*** | 21 |
| ***Apis dorsata*** | 20 |
| ***Drosophila erecta*** | 20 |
| ***Larimichthys crocea*** | 19 |
| ***Fopius arisanus*** | 19 |
| ***Bactrocera dorsalis*** | 19 |
| ***Omphisa fuscidentalis*** | 19 |
| ***Ceratosolen solmsi marchali*** | 18 |
| ***Antheraea yamamai*** | 17 |
| ***Ceratitis capitata*** | 16 |
| ***Bombus terrestris*** | 16 |
| ***Apis mellifera*** | 16 |
| ***Trichoplusia ni*** | 16 |
| ***Drosophila sechellia*** | 15 |
| ***Drosophila simulans*** | 15 |
| ***Helicoverpa assulta*** | 14 |
| ***Bacillus selenitireducens]*** | 14 |
| ***Choristoneura fumiferana*** | 14 |
| ***Ectropis obliqua*** | 14 |
| ***Helicoverpa zea*** | 13 |
| ***Heliothis virescens*** | 13 |
| ***Amphimedon queenslandica*** | 13 |
| ***Homo sapiens*** | 12 |
| ***Agrotis ipsilon*** | 12 |
| ***Bicyclus anynana*** | 12 |
| ***Clonorchis sinensis*** | 12 |
| ***Monomorium pharaonis*** | 11 |
| ***Bombus impatiens*** | 11 |
| ***Pectinophora gossypiella*** | 10 |
| ***Apis florea*** | 10 |
| ***Haemonchus contortus*** | 10 |
| ***Latimeria chalumnae*** | 10 |
| ***Ostrinia scapulalis*** | 10 |
| ***Trichuris suis*** | 10 |
| ***Danio rerio*** | 10 |
| ***Bombyx mandarina*** | 10 |
| ***Oesophagostomum dentatum*** | 10 |
| ***Xenopus (Silurana) tropicalis*** | 9 |
| ***Lonomia obliqua*** | 9 |
| ***Callimorpha dominula*** | 8 |
| ***Bactrocera cucurbitae*** | 8 |
| ***Glyptapanteles flavicoxis*** | 8 |
| ***Euphydryas aurinia*** | 8 |
| ***Thelohanellus kitauei*** | 8 |
| ***Aplysia californica*** | 8 |
| ***Nematostella vectensis*** | 7 |
| ***Mamestra brassicae*** | 7 |
| ***Junonia coenia*** | 7 |
| ***Cydia pomonella*** | 7 |
| ***Mizuhopecten yessoensis*** | 7 |
| ***Pogonomyrmex barbatus*** | 7 |
| ***Pieris rapae*** | 7 |
| ***Cricetulus griseus*** | 7 |
| ***Mythimna separata*** | 7 |
| ***Schistosoma japonicum*** | 7 |
| ***Brugia malayi*** | 7 |
| ***Leptopilina clavipes*** | 6 |
| ***Salmo salar*** | 6 |
| ***Mus musculus*** | 6 |
| ***Sesamia inferens*** | 6 |
| ***Chaetura pelagica*** | 6 |
| ***Coptotermes formosanus*** | 6 |
| ***Plodia interpunctella*** | 6 |
| ***Atta cephalotes*** | 6 |
| ***Esox lucius*** | 6 |
| ***Biomphalaria glabrata*** | 5 |
| ***Oeneis sculda*** | 5 |
| ***Rhinopithecus roxellana*** | 5 |
| ***Anas platyrhynchos*** | 5 |
| ***Schistosoma mansoni*** | 5 |
| ***Samia cynthia*** | 4 |
| ***Capitella teleta*** | 4 |
| ***Fundulus heteroclitus*** | 4 |
| ***Oryctolagus cuniculus*** | 4 |
| ***Oreochromis niloticus*** | 4 |
| ***Riptortus pedestris*** | 4 |
| ***Melitaea cinxia*** | 4 |
| ***Ostrinia penitalis*** | 4 |
| ***Glyptapanteles indiensis*** | 4 |
| ***Oryzias latipes*** | 4 |
| ***Rhodococcus*** | 4 |
| ***Diatraea saccharalis*** | 4 |
| ***Caenorhabditis elegans*** | 4 |
| ***Papilio dardanus*** | 4 |
| ***Ciona intestinalis*** | 4 |
| ***Gymnochlora stellata*** | 4 |
| ***Samia ricini*** | 3 |
| ***Glypta fumiferanae*** | 3 |
| ***Gallus gallus*** | 3 |
| ***Ancylostoma duodenale*** | 3 |
| ***Azumapecten farreri*** | 3 |
| ***Maylandia zebra*** | 3 |
| ***Bacillus selenitireducens MLS10]*** | 3 |
| ***Ovis aries musimon*** | 3 |
| ***Dendrolimus houi*** | 3 |
| ***Echinococcus granulosus*** | 3 |
| ***Sorex araneus*** | 3 |
| ***Plasmodium vivax Sal-1*** | 3 |
| ***Takifugu rubripes*** | 3 |
| ***Pararge aegeria*** | 3 |
| ***Portunus pelagicus*** | 3 |
| ***Hyphantria cunea*** | 3 |
| ***Conogethes punctiferalis*** | 3 |
| ***Caenorhabditis briggsae*** | 3 |
| ***Pantholops hodgsonii*** | 3 |
| ***Ceratitis rosa*** | 3 |
| ***Ancylostoma ceylanicum*** | 3 |
| ***Helobdella robusta*** | 3 |
| ***Callorhinchus milii*** | 3 |
| ***Myotis brandtii*** | 3 |
| ***Mya arenaria*** | 3 |
| ***Trichomonas vaginalis G3*** | 3 |
| ***Agrius convolvuli*** | 2 |
| ***Lepeophtheirus salmonis*** | 2 |
| ***Haemaphysalis longicornis*** | 2 |
| ***Notothenia coriiceps*** | 2 |
| ***Felis catus*** | 2 |
| ***Octodon degus*** | 2 |
| ***Calypte anna*** | 2 |
| ***Marsupenaeus japonicus*** | 2 |
| ***Eimeria necatrix*** | 2 |
| ***Trichoplax adhaerens*** | 2 |
| ***Plasmodium knowlesi strain H*** | 2 |
| ***Phengaris teleius*** | 2 |
| ***Maruca vitrata*** | 2 |
| ***Mytilus edulis*** | 2 |
| ***Poecilia formosa*** | 2 |
| ***Grapholita molesta*** | 2 |
| ***Heliothis subflexa*** | 2 |
| ***Plasmodiophora brassicae*** | 2 |
| ***Rattus norvegicus*** | 2 |
| ***Echinops telfairi*** | 2 |
| ***Odobenus rosmarus divergens*** | 2 |
| ***Heliconius numata silvana*** | 2 |
| ***Heliconius melpomene melpomene*** | 2 |
| ***Lipotes vexillifer*** | 2 |
| ***Xenopus laevis*** | 2 |
| ***Zygaena filipendulae*** | 2 |
| ***Capra hircus*** | 2 |
| ***Bigelowiella natans*** | 2 |
| ***Heliconius erato favorinus x Heliconius erato emma*** | 2 |
| ***Actias selene*** | 2 |
| ***Perkinsus marinus ATCC 50983*** | 2 |
| ***Alligator sinensis*** | 2 |
| ***Mythimna unipuncta*** | 2 |
| ***Dendroctonus ponderosae*** | 2 |
| ***Lymantria dispar*** | 2 |
| ***Heliconius erato favorinus*** | 2 |
| ***Hirudo medicinalis*** | 2 |
| ***Triatoma infestans*** | 2 |
| ***Toxocara canis*** | 2 |
| ***Anopheles stephensi*** | 2 |
| ***Drosophila buzzatii*** | 2 |
| ***Bos taurus*** | 2 |
| ***Bison bison bison*** | 2 |
| ***Bos mutus*** | 2 |
| ***Pilumnus hirtellus*** | 2 |
| ***Dendrolimus kikuchii*** | 2 |
| ***Corvus brachyrhynchos*** | 2 |
| ***Picoides pubescens*** | 1 |
| ***Plasmodium yoelii yoelii*** | 1 |
| ***Aglia tau*** | 1 |
| ***Ephestia kuehniella*** | 1 |
| ***Nomascus leucogenys*** | 1 |
| ***Lasiocampa quercus*** | 1 |
| ***Ophiophagus hannah*** | 1 |
| ***Manacus vitellinus*** | 1 |
| ***Orycteropus afer afer*** | 1 |
| ***Siniperca chuatsi*** | 1 |
| ***Fukomys damarensis*** | 1 |
| ***Papio anubis*** | 1 |
| ***Apis mellifera ligustica*** | 1 |
| ***Ctenopseustis herana*** | 1 |
| ***Drosophila pachea*** | 1 |
| ***Rhodnius prolixus*** | 1 |
| ***Arenicola marina*** | 1 |
| ***Shijimiaeoides divina*** | 1 |
| ***Camelus ferus*** | 1 |
| ***Tarsius syrichta*** | 1 |
| ***Eriphia verrucosa*** | 1 |
| ***Agrius cingulata*** | 1 |
| ***Heliconius melpomene aglaope*** | 1 |
| ***Pristionchus pacificus*** | 1 |
| ***Opisthocomus hoazin*** | 1 |
| ***Tirumala septentrionis*** | 1 |
| ***Heliconius melpomene xenoclea*** | 1 |
| ***Polygonia c-album*** | 1 |
| ***Nuttalliella namaqua*** | 1 |
| ***Stylonychia lemnae*** | 1 |
| ***Coenonympha glycerion*** | 1 |
| ***Oreta rosea*** | 1 |
| ***Podiceps cristatus*** | 1 |
| ***Cavia porcellus*** | 1 |
| ***Chilo auricilius*** | 1 |
| ***Leptosomus discolor*** | 1 |
| ***Dictyostelium discoideum AX4*** | 1 |
| ***Ostrinia narynensis*** | 1 |
| ***Haliaeetus leucocephalus*** | 1 |
| ***Neoceratodus forsteri*** | 1 |
| ***Heliconius numata aurora*** | 1 |
| ***Heliconius melpomene cythera*** | 1 |
| ***Planotortrix octo*** | 1 |
| ***Suberites domuncula*** | 1 |
| ***Acanthamoeba castellanii str. Neff*** | 1 |
| ***Ficedula albicollis*** | 1 |
| ***Drosophila americana*** | 1 |
| ***Caligus rogercresseyi*** | 1 |
| ***Chrysochloris asiatica*** | 1 |
| ***Anopheles funestus*** | 1 |
| ***Chironomus tentans*** | 1 |
| ***Xanthopan morgani praedicta*** | 1 |
| ***Dastarcus helophoroides*** | 1 |
| ***Poecilia reticulata*** | 1 |
| ***Pelecanus crispus*** | 1 |
| ***Nuclearia simplex*** | 1 |
| ***Amblyomma hebraeum*** | 1 |
| ***Colius striatus*** | 1 |
| ***Cuculus canorus*** | 1 |
| ***Adoxophyes honmai*** | 1 |
| ***Agrotis exclamationis*** | 1 |
| ***Anolis carolinensis*** | 1 |
| ***Naegleria gruberi*** | 1 |
| ***Nannospalax galili*** | 1 |
| ***Haplochromis burtoni*** | 1 |
| ***Gregarina niphandrodes*** | 1 |
| ***Taeniopygia guttata*** | 1 |
| ***Sesamia calamistis*** | 1 |
| ***Adineta vaga*** | 1 |
| ***Corcyra cephalonica*** | 1 |
| ***Echinococcus multilocularis*** | 1 |
| ***Daphnia pulex*** | 1 |
| ***Plasmodium reichenowi*** | 1 |
| ***Heliconius heurippa*** | 1 |
| ***Triuncina brunnea*** | 1 |
| ***Columba livia*** | 1 |
| ***Branchiostoma floridae*** | 1 |
| ***Ostrinia latipennis*** | 1 |
| ***Scoliopteryx libatrix*** | 1 |
| ***Eueides isabella*** | 1 |
| ***Plasmodium falciparum Tanzania (2000708)*** | 1 |
| ***Triatoma brasiliensis*** | 1 |
| ***Efflatouniella aegyptiaca*** | 1 |
| ***Heliconius numata*** | 1 |
| ***Ornithorhynchus anatinus*** | 1 |
| ***Schistocerca gregaria*** | 1 |
| ***Microplitis mediator*** | 1 |
| ***Abraxas sylvata*** | 1 |
| ***Heliothis viriplaca*** | 1 |
| ***Locusta migratoria*** | 1 |
| ***Phalera flavescens*** | 1 |
| ***Macaca nemestrina*** | 1 |
| ***Cathartes aura*** | 1 |
| ***Oxytricha trifallax*** | 1 |
| ***Dictyostelium purpureum*** | 1 |
| ***Erinaceus europaeus*** | 1 |
| ***Liriomyza huidobrensis*** | 1 |
| ***Nephila pilipes*** | 1 |
| ***Vanessa cardui*** | 1 |
| ***Pelodiscus sinensis*** | 1 |
| ***Epinephelus bruneus*** | 1 |
| ***Serinus canaria*** | 1 |
| ***Stegastes partitus*** | 1 |
| ***Chelonia mydas*** | 1 |
| ***Heliconius hewitsoni*** | 1 |
| ***Plasmodium falciparum 3D7*** | 1 |
| ***Dromaius novaehollandiae*** | 1 |
| ***Pacifastacus leniusculus*** | 1 |
| ***Lepisosteus oculatus*** | 1 |
| ***Gorilla gorilla*** | 1 |
| ***Scleropages formosus*** | 1 |
| ***Chlamydotis macqueenii*** | 1 |
| ***Periplaneta americana*** | 1 |
| ***Heliconius numata arcuella*** | 1 |
| ***Onthophagus binodis*** | 1 |
| ***Ceratomia catalpae*** | 1 |
| ***Jaculus jaculus*** | 1 |
| ***Caprimulgus carolinensis*** | 1 |
| ***Heliconius demeter*** | 1 |
| ***Ceratotherium simum simum*** | 1 |
| ***Galeopterus variegatus*** | 1 |
| ***Pristionchus sp. 15 RS5229*** | 1 |
| ***Cotesia rubecula*** | 1 |
| ***Nucula nucleus*** | 1 |
| ***Amblyomma americanum*** | 1 |
| ***Dudgeonea sp. Dudg*** | 1 |
| ***Drosophila teissieri*** | 1 |
| ***Fonticula alba*** | 1 |
| ***Bemisia tabaci*** | 1 |
| ***Chrysodeixis includens*** | 1 |
| ***Nilaparvata lugens*** | 1 |
| ***Liobuthus kessleri*** | 1 |
| ***Tinamus guttatus*** | 1 |
| ***Cherax quadricarinatus*** | 1 |
| ***Theileria parva strain Muguga*** | 1 |
| ***Chortoicetes terminifera*** | 1 |
| ***Cercocebus atys*** | 1 |
| ***Platynereis dumerilii*** | 1 |
| ***Gryllus bimaculatus*** | 1 |
| ***Cupiennius salei*** | 1 |
| ***Cotesia congregata*** | 1 |
| ***Trypanosoma cruzi*** | 1 |
| ***Merops nubicus*** | 1 |
| ***Balaenoptera acutorostrata scammoni*** | 1 |
| ***Corvus cornix cornix*** | 1 |
| ***Osmerus mordax*** | 1 |
| ***Thermobia domestica*** | 1 |
| ***Heliconius timareta timareta*** | 1 |
| ***Colobochyla salicalis*** | 1 |
| ***Caenorhabditis brenneri*** | 1 |
| ***Alabama argillacea*** | 1 |
| ***Samia cynthia walkeri*** | 1 |
| ***Haplopelma schmidti*** | 1 |
| ***Scolopendra polymorpha*** | 1 |
| ***Stomoxys calcitrans*** | 1 |
| ***Colias eurytheme*** | 1 |
| ***Ptychoglene phrada*** | 1 |

**Table S2 The List of Pathway genes**

| **KEGG_A_class** | **KEGG_B_class** | **#Pathway** | **Count (8000)** |
| --- | --- | --- | --- |
| **Metabolism** | Global and overview maps | Metabolic pathways | 1364 |
| **Human Diseases** | Cancers: Overview | Pathways in cancer | 277 |
| **Genetic Information Processing** | Translation | RNA transport | 243 |
| **Metabolism** | Nucleotide metabolism | Purine metabolism | 240 |
| **Genetic Information Processing** | Folding, sorting and degradation | Protein processing in endoplasmic reticulum | 235 |
| **Genetic Information Processing** | Folding, sorting and degradation | Ubiquitin mediated proteolysis | 224 |
| **Genetic Information Processing** | Transcription | Spliceosome | 207 |
| **Human Diseases** | Neurodegenerative diseases | Huntington's disease | 206 |
| **Cellular Processes** | Cellular community | Focal adhesion | 191 |
| **Human Diseases** | Neurodegenerative diseases | Alzheimer's disease | 182 |
| **Cellular Processes** | Transport and catabolism | Endocytosis | 172 |
| **Cellular Processes** | Cell growth and death | Cell cycle | 170 |
| **Metabolism** | Nucleotide metabolism | Pyrimidine metabolism | 169 |
| **Cellular Processes** | Cell motility | Regulation of actin cytoskeleton | 160 |
| **Environmental Information Processing** | Signal transduction | MAPK signaling pathway | 159 |
| **Cellular Processes** | Transport and catabolism | Lysosome | 155 |
| **Organismal Systems** | Endocrine system | Insulin signaling pathway | 146 |
| **Metabolism** | Energy metabolism | Oxidative phosphorylation | 141 |
| **Cellular Processes** | Transport and catabolism | Peroxisome | 139 |
| **Environmental Information Processing** | Signal transduction | Wnt signaling pathway | 138 |
| **Human Diseases** | Infectious diseases: Viral | Influenza A | 137 |
| **Human Diseases** | Neurodegenerative diseases | Parkinson's disease | 137 |
| **Genetic Information Processing** | Translation | mRNA surveillance pathway | 123 |
| **Genetic Information Processing** | Translation | Ribosome biogenesis in eukaryotes | 122 |
| **Cellular Processes** | Cell growth and death | Oocyte meiosis | 118 |
| **Organismal Systems** | Immune system | Chemokine signaling pathway | 112 |
| **Cellular Processes** | Cellular community | Tight junction | 112 |
| **Genetic Information Processing** | Translation | Ribosome | 111 |
| **Human Diseases** | Cancers: Specific types | Prostate cancer | 106 |
| **Organismal Systems** | Digestive system | Pancreatic secretion | 106 |
| **Organismal Systems** | Digestive system | Protein digestion and absorption | 104 |
| **Organismal Systems** | Digestive system | Bile secretion | 102 |
| **Genetic Information Processing** | Folding, sorting and degradation | RNA degradation | 101 |
| **Environmental Information Processing** | Signal transduction | Calcium signaling pathway | 100 |
| **Metabolism** | Xenobiotics biodegradation and metabolism | Drug metabolism - other enzymes | 96 |
| **Organismal Systems** | Circulatory system | Vascular smooth muscle contraction | 91 |
| **Organismal Systems** | Endocrine system | Progesterone-mediated oocyte maturation | 91 |
| **Metabolism** | Metabolism of other amino acids | Glutathione metabolism | 90 |
| **Genetic Information Processing** | Replication and repair | Nucleotide excision repair | 88 |
| **Environmental Information Processing** | Signaling molecules and interaction | ECM-receptor interaction | 87 |
| **Human Diseases** | Infectious diseases: Parasitic | Amoebiasis | 86 |
| **Organismal Systems** | Endocrine system | GnRH signaling pathway | 86 |
| **Organismal Systems** | Development | Axon guidance | 85 |
| **Cellular Processes** | Transport and catabolism | Phagosome | 85 |
| **Organismal Systems** | Nervous system | Neurotrophin signaling pathway | 85 |
| **Metabolism** | Lipid metabolism | Glycerophospholipid metabolism | 84 |
| **Environmental Information Processing** | Signal transduction | ErbB signaling pathway | 83 |
| **Genetic Information Processing** | Replication and repair | DNA replication | 83 |
| **Organismal Systems** | Digestive system | Mineral absorption | 82 |
| **Cellular Processes** | Cellular community | Adherens junction | 82 |
| **Organismal Systems** | Endocrine system | PPAR signaling pathway | 81 |
| **Genetic Information Processing** | Translation | Aminoacyl-tRNA biosynthesis | 81 |
| **Human Diseases** | Cardiovascular diseases | Dilated cardiomyopathy | 81 |
| **Organismal Systems** | Endocrine system | Melanogenesis | 80 |
| **Environmental Information Processing** | Signal transduction | Phosphatidylinositol signaling system | 79 |
| **Metabolism** | Carbohydrate metabolism | Amino sugar and nucleotide sugar metabolism | 78 |
| **Human Diseases** | Infectious diseases: Parasitic | Toxoplasmosis | 78 |
| **Metabolism** | Amino acid metabolism | Lysine degradation | 78 |
| **Metabolism** | Amino acid metabolism | Valine, leucine and isoleucine degradation | 76 |
| **Human Diseases** | Infectious diseases: Bacterial | Bacterial invasion of epithelial cells | 75 |
| **Metabolism** | Glycan biosynthesis and metabolism | N-Glycan biosynthesis | 75 |
| **Human Diseases** | Cardiovascular diseases | Hypertrophic cardiomyopathy (HCM) | 73 |
| **Organismal Systems** | Digestive system | Salivary secretion | 72 |
| **Environmental Information Processing** | Signal transduction | mTOR signaling pathway | 71 |
| **Cellular Processes** | Cellular community | Gap junction | 70 |
| **Environmental Information Processing** | Membrane transport | ABC transporters | 70 |
| **Metabolism** | Xenobiotics biodegradation and metabolism | Metabolism of xenobiotics by cytochrome P450 | 69 |
| **Organismal Systems** | Immune system | Fc gamma R-mediated phagocytosis | 69 |
| **Metabolism** | Carbohydrate metabolism | Starch and sucrose metabolism | 69 |
| **Organismal Systems** | Circulatory system | Cardiac muscle contraction | 68 |
| **Human Diseases** | Infectious diseases: Bacterial | Tuberculosis | 68 |
| **Human Diseases** | Infectious diseases: Viral | Measles | 68 |
| **Organismal Systems** | Digestive system | Vitamin digestion and absorption | 66 |
| **Human Diseases** | Cancers: Specific types | Small cell lung cancer | 66 |
| **Metabolism** | Carbohydrate metabolism | Inositol phosphate metabolism | 65 |
| **Human Diseases** | Infectious diseases: Bacterial | Shigellosis | 65 |
| **Human Diseases** | Cancers: Specific types | Renal cell carcinoma | 64 |
| **Organismal Systems** | Immune system | Leukocyte transendothelial migration | 64 |
| **Environmental Information Processing** | Signaling molecules and interaction | Neuroactive ligand-receptor interaction | 64 |
| **Metabolism** | Amino acid metabolism | Arginine and proline metabolism | 64 |
| **Metabolism** | Lipid metabolism | Glycerolipid metabolism | 64 |
| **Metabolism** | Lipid metabolism | Fatty acid metabolism | 63 |
| **Metabolism** | Xenobiotics biodegradation and metabolism | Drug metabolism - cytochrome P450 | 62 |
| **Organismal Systems** | Immune system | T cell receptor signaling pathway | 62 |
| **Human Diseases** | Cancers: Specific types | Glioma | 62 |
| **Organismal Systems** | Nervous system | Glutamatergic synapse | 61 |
| **Organismal Systems** | Digestive system | Gastric acid secretion | 60 |
| **Organismal Systems** | Nervous system | Cholinergic synapse | 59 |
| **Metabolism** | Carbohydrate metabolism | Pyruvate metabolism | 59 |
| **Metabolism** | Carbohydrate metabolism | Pentose and glucuronate interconversions | 58 |
| **Human Diseases** | Infectious diseases: Bacterial | Epithelial cell signaling in Helicobacter pylori infection | 58 |
| **Genetic Information Processing** | Transcription | RNA polymerase | 58 |
| **Organismal Systems** | Nervous system | Long-term potentiation | 57 |
| **Environmental Information Processing** | Signal transduction | TGF-beta signaling pathway | 57 |
| **Human Diseases** | Infectious diseases: Viral | Hepatitis C | 56 |
| **Metabolism** | Carbohydrate metabolism | Glycolysis / Gluconeogenesis | 56 |
| **Organismal Systems** | Endocrine system | Adipocytokine signaling pathway | 56 |
| **Metabolism** | Carbohydrate metabolism | Citrate cycle (TCA cycle) | 56 |
| **Metabolism** | Amino acid metabolism | Alanine, aspartate and glutamate metabolism | 55 |
| **Environmental Information Processing** | Signal transduction | Hedgehog signaling pathway | 54 |
| **Metabolism** | Carbohydrate metabolism | Galactose metabolism | 54 |
| **Human Diseases** | Infectious diseases: Bacterial | Vibrio cholerae infection | 54 |
| **Organismal Systems** | Sensory system | Phototransduction - fly | 54 |
| **Genetic Information Processing** | Replication and repair | Base excision repair | 53 |
| **Human Diseases** | Infectious diseases: Bacterial | Pathogenic Escherichia coli infection | 52 |
| **Human Diseases** | Infectious diseases: Parasitic | Chagas disease (American trypanosomiasis) | 51 |
| **Genetic Information Processing** | Transcription | Basal transcription factors | 51 |
| **Metabolism** | Metabolism of other amino acids | beta-Alanine metabolism | 51 |
| **Human Diseases** | Cancers: Specific types | Endometrial cancer | 51 |
| **Cellular Processes** | Cell growth and death | p53 signaling pathway | 50 |
| **Metabolism** | Carbohydrate metabolism | Propanoate metabolism | 49 |
| **Human Diseases** | Cardiovascular diseases | Arrhythmogenic right ventricular cardiomyopathy (ARVC) | 49 |
| **Genetic Information Processing** | Folding, sorting and degradation | Proteasome | 49 |
| **Human Diseases** | Cancers: Specific types | Acute myeloid leukemia | 48 |
| **Organismal Systems** | Immune system | Fc epsilon RI signaling pathway | 48 |
| **Environmental Information Processing** | Signal transduction | Jak-STAT signaling pathway | 48 |
| **Organismal Systems** | Excretory system | Vasopressin-regulated water reabsorption | 48 |
| **Organismal Systems** | Excretory system | Endocrine and other factor-regulated calcium reabsorption | 47 |
| **Human Diseases** | Neurodegenerative diseases | Amyotrophic lateral sclerosis (ALS) | 47 |
| **Human Diseases** | Cancers: Specific types | Basal cell carcinoma | 47 |
| **Human Diseases** | Cancers: Specific types | Chronic myeloid leukemia | 47 |
| **Metabolism** | Metabolism of cofactors and vitamins | Porphyrin and chlorophyll metabolism | 46 |
| **Organismal Systems** | Development | Osteoclast differentiation | 46 |
| **Organismal Systems** | Development | Dorso-ventral axis formation | 46 |
| **Organismal Systems** | Endocrine system | Renin-angiotensin system | 46 |
| **Environmental Information Processing** | Signal transduction | MAPK signaling pathway - fly | 46 |
| **Environmental Information Processing** | Signal transduction | Notch signaling pathway | 45 |
| **Human Diseases** | Endocrine and metabolic diseases | Type II diabetes mellitus | 45 |
| **Organismal Systems** | Immune system | Natural killer cell mediated cytotoxicity | 44 |
| **Metabolism** | Carbohydrate metabolism | Fructose and mannose metabolism | 44 |
| **Metabolism** | Amino acid metabolism | Cysteine and methionine metabolism | 42 |
| **Genetic Information Processing** | Replication and repair | Homologous recombination | 42 |
| **Human Diseases** | Cancers: Specific types | Colorectal cancer | 42 |
| **Metabolism** | Amino acid metabolism | Glycine, serine and threonine metabolism | 41 |
| **Human Diseases** | Cardiovascular diseases | Viral myocarditis | 41 |
| **Environmental Information Processing** | Signal transduction | VEGF signaling pathway | 41 |
| **Organismal Systems** | Immune system | B cell receptor signaling pathway | 40 |
| **Human Diseases** | Neurodegenerative diseases | Prion diseases | 40 |
| **Organismal Systems** | Immune system | Antigen processing and presentation | 40 |
| **Metabolism** | Lipid metabolism | Biosynthesis of unsaturated fatty acids | 39 |
| **Organismal Systems** | Digestive system | Fat digestion and absorption | 39 |
| **Environmental Information Processing** | Signaling molecules and interaction | Cell adhesion molecules (CAMs) | 39 |
| **Metabolism** | Metabolism of cofactors and vitamins | Retinol metabolism | 38 |
| **Metabolism** | Carbohydrate metabolism | Pentose phosphate pathway | 37 |
| **Cellular Processes** | Cell growth and death | Apoptosis | 37 |
| **Human Diseases** | Cancers: Specific types | Pancreatic cancer | 37 |
| **Organismal Systems** | Immune system | Cytosolic DNA-sensing pathway | 36 |
| **Genetic Information Processing** | Replication and repair | Mismatch repair | 36 |
| **Organismal Systems** | Digestive system | Carbohydrate digestion and absorption | 36 |
| **Organismal Systems** | Immune system | Toll-like receptor signaling pathway | 35 |
| **Metabolism** | Glycan biosynthesis and metabolism | Other types of O-glycan biosynthesis | 35 |
| **Metabolism** | Lipid metabolism | Sphingolipid metabolism | 34 |
| **Organismal Systems** | Immune system | Hematopoietic cell lineage | 34 |
| **Metabolism** | Lipid metabolism | Steroid hormone biosynthesis | 34 |
| **Metabolism** | Carbohydrate metabolism | Ascorbate and aldarate metabolism | 34 |
| **Organismal Systems** | Nervous system | Long-term depression | 34 |
| **Human Diseases** | Cancers: Specific types | Non-small cell lung cancer | 33 |
| **Metabolism** | Glycan biosynthesis and metabolism | Glycosylphosphatidylinositol(GPI)-anchor biosynthesis | 33 |
| **Metabolism** | Amino acid metabolism | Tryptophan metabolism | 32 |
| **Organismal Systems** | Excretory system | Proximal tubule bicarbonate reclamation | 31 |
| **Genetic Information Processing** | Folding, sorting and degradation | SNARE interactions in vesicular transport | 31 |
| **Metabolism** | Glycan biosynthesis and metabolism | Glycosaminoglycan degradation | 29 |
| **Metabolism** | Metabolism of terpenoids and polyketides | Terpenoid backbone biosynthesis | 29 |
| **Genetic Information Processing** | Folding, sorting and degradation | Protein export | 28 |
| **Metabolism** | Carbohydrate metabolism | Butanoate metabolism | 28 |
| **Metabolism** | Carbohydrate metabolism | Glyoxylate and dicarboxylate metabolism | 26 |
| **Metabolism** | Lipid metabolism | alpha-Linolenic acid metabolism | 26 |
| **Human Diseases** | Infectious diseases: Bacterial | Pertussis | 25 |
| **Human Diseases** | Infectious diseases: Parasitic | Leishmaniasis | 25 |
| **Metabolism** | Amino acid metabolism | Tyrosine metabolism | 25 |
| **Metabolism** | Lipid metabolism | Arachidonic acid metabolism | 25 |
| **Metabolism** | Lipid metabolism | Fatty acid biosynthesis | 24 |
| **Metabolism** | Glycan biosynthesis and metabolism | Other glycan degradation | 24 |
| **Human Diseases** | Immune diseases | Rheumatoid arthritis | 24 |
| **Organismal Systems** | Immune system | RIG-I-like receptor signaling pathway | 23 |
| **Metabolism** | Metabolism of cofactors and vitamins | Folate biosynthesis | 23 |
| **Metabolism** | Glycan biosynthesis and metabolism | Glycosaminoglycan biosynthesis - heparan sulfate | 23 |
| **Human Diseases** | Cancers: Specific types | Melanoma | 22 |
| **Metabolism** | Lipid metabolism | Linoleic acid metabolism | 22 |
| **Organismal Systems** | Immune system | NOD-like receptor signaling pathway | 22 |
| **Organismal Systems** | Excretory system | Collecting duct acid secretion | 22 |
| **Human Diseases** | Cancers: Specific types | Thyroid cancer | 22 |
| **Organismal Systems** | Excretory system | Aldosterone-regulated sodium reabsorption | 22 |
| **Metabolism** | Lipid metabolism | Ether lipid metabolism | 22 |
| **Metabolism** | Amino acid metabolism | Phenylalanine metabolism | 22 |
| **Organismal Systems** | Sensory system | Olfactory transduction | 22 |
| **Organismal Systems** | Environmental adaptation | Circadian rhythm - mammal | 21 |
| **Metabolism** | Metabolism of cofactors and vitamins | Nicotinate and nicotinamide metabolism | 21 |
| **Metabolism** | Metabolism of cofactors and vitamins | One carbon pool by folate | 21 |
| **Human Diseases** | Immune diseases | Systemic lupus erythematosus | 20 |
| **Cellular Processes** | Transport and catabolism | Regulation of autophagy | 20 |
| **Metabolism** | Biosynthesis of other secondary metabolites | Caffeine metabolism | 20 |
| **Organismal Systems** | Environmental adaptation | Circadian rhythm - fly | 20 |
| **Metabolism** | Lipid metabolism | Fatty acid elongation in mitochondria | 20 |
| **Metabolism** | Metabolism of terpenoids and polyketides | Insect hormone biosynthesis | 19 |
| **Metabolism** | Metabolism of other amino acids | Selenocompound metabolism | 19 |
| **Metabolism** | Metabolism of cofactors and vitamins | Ubiquinone and other terpenoid-quinone biosynthesis | 19 |
| **Environmental Information Processing** | Signaling molecules and interaction | Cytokine-cytokine receptor interaction | 17 |
| **Metabolism** | Metabolism of cofactors and vitamins | Pantothenate and CoA biosynthesis | 16 |
| **Human Diseases** | Cancers: Specific types | Bladder cancer | 16 |
| **Metabolism** | Amino acid metabolism | Histidine metabolism | 15 |
| **Genetic Information Processing** | Replication and repair | Non-homologous end-joining | 15 |
| **Organismal Systems** | Sensory system | Taste transduction | 14 |
| **Metabolism** | Glycan biosynthesis and metabolism | Glycosaminoglycan biosynthesis - chondroitin sulfate | 13 |
| **Metabolism** | Glycan biosynthesis and metabolism | Mucin type O-Glycan biosynthesis | 12 |
| **Human Diseases** | Infectious diseases: Parasitic | Malaria | 11 |
| **Genetic Information Processing** | Folding, sorting and degradation | Sulfur relay system | 11 |
| **Metabolism** | Glycan biosynthesis and metabolism | Glycosphingolipid biosynthesis - globo series | 11 |
| **Metabolism** | Amino acid metabolism | Valine, leucine and isoleucine biosynthesis | 11 |
| **Metabolism** | Metabolism of other amino acids | Taurine and hypotaurine metabolism | 10 |
| **Human Diseases** | Infectious diseases: Parasitic | African trypanosomiasis | 10 |
| **Metabolism** | Metabolism of cofactors and vitamins | Riboflavin metabolism | 9 |
| **Metabolism** | Lipid metabolism | Steroid biosynthesis | 8 |
| **Metabolism** | Amino acid metabolism | Phenylalanine, tyrosine and tryptophan biosynthesis | 8 |
| **Human Diseases** | Endocrine and metabolic diseases | Maturity onset diabetes of the young | 8 |
| **Metabolism** | Lipid metabolism | Primary bile acid biosynthesis | 7 |
| **Metabolism** | Metabolism of other amino acids | D-Glutamine and D-glutamate metabolism | 7 |
| **Human Diseases** | Endocrine and metabolic diseases | Type I diabetes mellitus | 7 |
| **Metabolism** | Lipid metabolism | Synthesis and degradation of ketone bodies | 7 |
| **Metabolism** | Glycan biosynthesis and metabolism | Glycosphingolipid biosynthesis - ganglio series | 6 |
| **Human Diseases** | Immune diseases | Primary immunodeficiency | 6 |
| **Metabolism** | Energy metabolism | Sulfur metabolism | 6 |
| **Metabolism** | Glycan biosynthesis and metabolism | Glycosphingolipid biosynthesis - lacto and neolacto series | 5 |
| **Metabolism** | Metabolism of cofactors and vitamins | Thiamine metabolism | 5 |
| **Organismal Systems** | Immune system | Complement and coagulation cascades | 5 |
| **Metabolism** | Metabolism of other amino acids | Cyanoamino acid metabolism | 5 |
| **Metabolism** | Biosynthesis of other secondary metabolites | Butirosin and neomycin biosynthesis | 5 |
| **Metabolism** | Glycan biosynthesis and metabolism | Glycosaminoglycan biosynthesis - keratan sulfate | 4 |
| **Organismal Systems** | Sensory system | Phototransduction | 4 |
| **Metabolism** | Metabolism of cofactors and vitamins | Vitamin B6 metabolism | 4 |
| **Metabolism** | Metabolism of cofactors and vitamins | Lipoic acid metabolism | 3 |
| **Metabolism** | Metabolism of cofactors and vitamins | Biotin metabolism | 3 |
| **Metabolism** | Metabolism of other amino acids | D-Arginine and D-ornithine metabolism | 2 |
| **Organismal Systems** | Immune system | Intestinal immune network for IgA production | 2 |
| **Metabolism** | Amino acid metabolism | Lysine biosynthesis | 1 |
| **Human Diseases** | Infectious diseases: Bacterial | Staphylococcus aureus infection | 1 |

Table S3 The top 10 up or down-regulated genes identified among eggs vs larvae, larvae vs pupae, and pupae vs adults.

| **GeneID** | **Source** | **Description** | **Eggs** | **Larvae** | **Pupae** | **Adults** | **Putative function** |
| --- | --- | --- | --- | --- | --- | --- | --- |
| Unigene0018273 | Eggs | proteoglycan 4-like [*Plutella xylostella*] | 101.92 | 0 | 0.20 | 0 | Joint lubrication and synovial homeostasis |
| Unigene0020554 | Eggs | pair-rule protein odd-paired-like [*Bombyx mori*] | 37.42 | 0 | 0.31 | 0.06 | **Development of the segmented embryos of insects** (KX450333) |
| Unigene0018275 | Eggs | leucine-rich repeat extensin-like protein 5 [*Bombyx mori*] | 37.21 | 0 | 0 | 0 | UNKNOWN |
| Unigene0002492 | Eggs | mucin-2-like [*Plutella xylostella*] | 32.64 | 0 | 343.99 | 0 | UNKNOWN |
| Unigene0013331 | Eggs | glucose dehydrogenase [*Danaus plexippus*] | 32.60 | 0 | 0.03 | 1.03 | UNKNOWN |
| Unigene0012958 | Eggs | epidermal growth factor receptor [*Bicyclus anynana*] | 31.90 | 0 | 0 | 0 | **Epidermal growth** (KX450334) |
| Unigene0028727 | Eggs | histone 2A variant [*Spodoptera frugiperda*] | 29.41 | 0 | 0.07 | 0 | DNA Folding |
| Unigene0011488 | Eggs | segmentation protein fushi tarazu [*Plutella xylostella*] | 27.89 | 0 | 0.04 | 0.02 | **Process of body segmentation**(KX450335) |
| Unigene0027414 | Eggs | hypothetical protein KGM_13352 [Danaus plexippus] | 26.79 | 0 | 0 | 0 | UNKNOWN |
| Unigene0015309 | Eggs | unknown secreted protein, partial [*Papilio xuthus*] | 19.68 | 0 | 0 | 0 | UNKNOWN |
| Unigene0041068 | Larvae | similar to CG12017 [*Papilio xuthus*] | 0 | 148.63 | 0 | 0.15 | UNKNOWN |
| Unigene0018912 | Larvae | uncharacterized protein [*Plutella xylostella*] | 0 | 149.99 | 0 | 0.10 | UNKNOWN |
| Unigene0026727 | Larvae | cuticular protein RR-1 [*Bombyx mori*] | 0 | 167.86 | 0.58 | 0 | **Cuticle sclerotization and adult molt** (KX450336) |
| Unigene0035947 | Larvae | hypothetical protein KGM_22714 [*Danaus plexippus*] | 0 | 173.97 | 0.73 | 1.83 | UNKNOWN |
| Unigene0001704 | Larvae | salivary cysteine-rich peptide precursor [*Bombyx mori*] | 0 | 234.13 | 1260.51 | 30.01 | UNKNOWN |
| Unigene0014429 | Larvae | unknown secreted protein [*Papilio polytes*] | 0 | 262.17 | 1.39 | 3.72 | UNKNOWN |
| Unigene0023331 | Larvae | mucin-5AC-like [*Bombyx mori*] | 0 | 293.94 | 177.43 | 55.86 | UNKNOWN |
| Unigene0006956 | Larvae | serine protease [*Bombyx mandarina*] | 0 | 333.90 | 1079.55 | 1.04 | UNKNOWN |
| Unigene0000597 | Larvae | GJ14248 [*Drosophila virilis*] | 0 | 749.23 | 0.77 | 0 | UNKNOWN |
| Unigene0033676 | Larvae | neuropeptide-like 4 [*Bombyx mori*] | 0 | 780.75 | 1032.64 | 2319.43 | UNKNOWN |
| Unigene0018912 | Larvae | uncharacterized protein [*Plutella xylostella*] | 0 | 149.99 | 0 | 0.10 | UNKNOWN |
| Unigene0041068 | Larvae | similar to CG12017 [*Papilio xuthus*] | 0 | 148.63 | 0 | 0.15 | UNKNOWN |
| Unigene0039166 | Larvae | peritrophin type-A protein 3 [*Danaus plexippus*] | 0.15 | 123.86 | 0 | 0 | Digestion |
| Unigene0027451 | Larvae | glucose dehydrogenase [*Bombyx mori*] | 0.95 | 67.36 | 0 | 5.16 | Catalyzes the chemical reaction |
| Unigene0027639 | Larvae | PREDICTED: formin-J-like [*Plutella xylostella*] | 0.59 | 51.77 | 0 | 0.45 | Controls junctional actin assembly and turnover |
| Unigene0026411 | Larvae | flocculation protein FLO11 [*Bombyx mori*] | 28.13 | 50.70 | 0 | 0.74 | Adhesion of the cells |
| Unigene0001809 | Larvae | seminal fluid protein CSSFP002 [*Chilo suppressalis*] | 0.30 | 48.17 | 0 | 0 | Reproductive function |
| Unigene0029121 | Larvae | Collagen alpha-2(I) chain, partial [Columba livia] | 0.0885 | 46.15 | 0 | 0.14 | UNKNOWN |
| Unigene0041857 | Larvae | PREDICTED: mucin-17 [*Plutella xylostella*] | 2.0 | 40.30 | 0 | 0.08 | Provide cytoprotection |
| Unigene0021007 | Larvae | glucose dehydrogenase [FAD, quinone] [*Bombyx mori*] | 0.28 | 39.49 | 0 | 0.03 | Catalyzes the chemical reaction |
| Unigene0020870 | Pupae | uncharacterized protein [*Bombyx mori*] | 0.07 | 0 | 509.60 | 0 | UNKNOWN |
| Unigene0030409 | Pupae | adhesive plaque matrix protein-like [*Saccoglossus kowalevskii*] | 0.10 | 0 | 648.75 | 0 | Adhesive function |
| Unigene0023404 | Pupae | collagen alpha-5(IV) chain-like [*Monomorium pharaonis*] | 0.56 | 0 | 702.52 | 0.24 | UNKNOWN |
| Unigene0036173 | Pupae | PREDICTED: mucin-19-like [Linepithema humile] | 0 | 0 | 765.23 | 0 | Mucus secretion |
| Unigene0016596 | Pupae | zonadhesin-like isoform X2 [*Bombyx mori*] | 6.83 | 0 | 840.24 | 944.93 | Sperm adhesion to the egg zona pellucida |
| Unigene0010258 | Pupae | MG17 [*Spodoptera exigua*] | 0 | 0 | 909.10 | 1.66 | UNKNOWN |
| Unigene0017382 | Pupae | Niemann-Pick type C2 protein [*Helicoverpa armigera*] | 0.35 | 0 | 1065.46 | 0 | Delivers cholesterol to the perimeter membrane |
| Unigene0036342 | Pupae | trypsin-like serine protease [*Ostrinia nubilalis*] | 0.10 | 0 | 1182.58 | 0 | UNKNOWN |
| Unigene0032550 | Pupae | chemosensory protein [*Cnaphalocrocis medinalis*] | 1.16 | 0 | 1792.30 | 1440.37 | **Larva developments, olfactory and gustatory function** (KX450337) |
| Unigene0016506 | Pupae | larval cuticle protein LCP-22 precursor [Bombyx mori] | 0 | 0 | 2383.60 | 0.06 | **Accumulates in integuments in larvae development** (KX450338) |
| Unigene0001330 | Pupae | larval cuticle protein LCP-17 precursor [*Bombyx mori*] | 0.27 | 0.96 | 8503.98 | 0 | **Accumulates in integuments in larvae development** (KX450339) |
| Unigene0030337 | Pupae | GL14840 [*Drosophila persimilis*] | 0.42 | 0.11 | 5165.32 | 0 | UNKNOWN |
| Unigene0035339 | Pupae | chitin deacetylase 2 [*Mamestra configurata*] | 3.18 | 0.56 | 3802.26 | 0 | **Delay in developmental timing** **(KX450340)** |
| Unigene0001331 | Pupae | larval cuticle protein LCP-17 precursor [*Bombyx mori*] | 0.16 | 0.22 | 3097.36 | 0 | **Accumulates in integuments in larvae development** (KX450341) |
| Unigene0001492 | Pupae | chymotrypsin-like serine protease [*Ostrinia nubilalis*] | 6.38 | 0.25 | 2472.20 | 0 | Necessary for the transcription of RNA |
| Unigene0030408 | Pupae | pancreatic triacylglycerol lipase-like [*Bombyx mori*] | 0.07 | 0.08 | 1739.23 | 0 | Lipid degradation |
| Unigene0001705 | Pupae | trypsin serine protease [*Ostrinia furnacalis*] | 1.06 | 0.19 | 1512.58 | 0 | UNKNOWN |
| Unigene0035700 | Pupae | lipase [*Helicoverpa armigera*] | 0.28 | 0.47 | 1470.38 | 0 | Essential roles in the digestion |
| Unigene0036342 | Pupae | trypsin-like serine protease [*Ostrinia nubilalis*] | 0.10 | 0 | 1182.58 | 0 | UNKNOWN |
| Unigene0031863 | Pupae | PREDICTED: mucin-19-like [*Linepithema humile*] | 0.06 | 0.08 | 1156.86 | 0 | Mucus secretion |
| Unigene0040326 | Adults | nose resistant to fluoxetine protein 6-like [*Plutella xylostella*] | 0 | 0.07 | 0 | 21.53 | **Multicellular organism development in larvae development** (KX450342) |
| Unigene0002921 | Adults | nuclear-pore anchor-like isoform X1 [*Bombyx mori*] | 0.03 | 51.87 | 0.06 | 1277.18 | Involved in mRNA export, telomere organization, spindle pole assembly |
| Unigene0002693 | Adults | insect intestinal mucin 4 [*Danaus plexippus*] | 0 | 1.78 | 0 | 34.20 | Associated with the peritrophic matrix |
| Unigene0012689 | Adults | retinol-binding protein [*Papilio xuthus*] | 0 | 3.28 | 0 | 52.33 | **Relative to development and growth of a conceptus** (KX450343) |
| Unigene0021965 | Adults | chitin binding domain 3 protein [*Mamestra configurata*] | 2.03 | 6.61 | 0 | 58.45 | **Relative to insect development** (KX450344) |
| Unigene0025688 | Adults | uncharacterized protein LOC105384628 [*Plutella xylostella*] | 0 | 0 | 0 | 107.98 | UNKNOWN |
| Unigene0030513 | Adults | uncharacterized protein LOC105388107 [*Plutella xylostella*] | 0 | 0 | 0 | 243.24 | UNKNOWN |
| Unigene0015076 | Adults | general odorant-binding protein 3 [*Cnaphalocrocis medinalis*] | 1.11 | 0 | 0 | 271.04 | Transmit odorant to the receptors |
| Unigene0027511 | Adults | probable ATP-dependent helicase PF08_0048 [*Plutella xylostella*] | 0.37 | 0.03 | 0 | 600.38 | UNKNOWN |
| Unigene0016473 | Adults | troponin C, isoform 1-like [*Plutella xylostella*] | 1.97 | 0.14 | 0 | 11493 | Increases power output and efficiency of muscles |

Different color showed the RPKM value in each tissue.

Indicated the RPKM value less than 5; indicated the RPKM value between 6 to 50; indicated the RPKM value between 51 to 100;

Indicated the RPKM value between 101 to 150 indicated the RPKM value between 151 to 200; indicated the RPKM value more than 200;

The putative functions related to insect development were in Bold display.
